# HIV Infection in Portugal: Measuring the Time Between Linkage to Care and Antiretroviral Therapy Initiation

**DOI:** 10.3390/healthcare13151812

**Published:** 2025-07-25

**Authors:** Ana Virgolino, Carolina Ferraz, Vanessa Nicolau, Rui Cortes, Aida Pereira, Fernando Maltez, João Paulo Caldas, João Lourinho, Maria Alves, Inês Caetano, Tiago Teixeira, Isabel Casella, Cristina Soeiro, Andreia Meseiro, Raquel Pinho, Andreia Ribeiro, Rosário Serrão, Francisco Antunes, on behalf of the Portuguese HIV Study Group

**Affiliations:** 1 Instituto de Saúde Ambiental, Faculdade de Medicina, Universidade de Lisboa, 1649-028 Lisboa, Portugal; nicolau.van@gmail.com (V.N.); fmaltez@chlc.min-saude.pt (F.M.); fantunes@medicina.ulisboa.pt (F.A.); 2 Lean Health Portugal, 1749-016 Lisboa, Portugal; carolina.ferraz@leanhealth.education (C.F.); lean@leanhealth.education (R.C.); 3 NOVA National School of Public Health, Public Health Research Center, Comprehensive Health Research Center, CHRC, NOVA University Lisbon, 1600-560 Lisboa, Portugal; 4 Serviço de Doenças Infeciosas, Centro Hospitalar Universitário Lisboa Norte (CHULN), EPE, Hospital de Santa Maria, 1649-028 Lisboa, Portugal; aida.valente@chln.min-saude.pt; 5 Serviço de Doenças Infeciosas, Centro Hospitalar Universitário Lisboa Central (CHULC), EPE, Hospital de Curry Cabral, 1069-066 Lisboa, Portugal; 6 Laboratório Associado TERRA, Faculdade de Medicina, Universidade de Lisboa, 1649-028 Lisboa, Portugal; 7 Serviço de Doenças Infeciosas, Unidade Local de Saúde São João, 4200-319 Porto, Portugalrosarioserrao@ulssjoao.min-saude.pt (R.S.); 8 Serviço de Infeciologia, Hospital Garcia de Orta, EPE, 2805-267 Almada, Portugal; joao.lourinho@hgo.min-saude.pt; 9 Serviço de Doenças Infeciosas, Hospital Professor Doutor Fernando da Fonseca, EPE, 2720-276 Amadora, Portugal; maria.alves@hff.min-saude.pt; 10 Serviço de Doenças Infeciosas, Unidade Local de Saúde de Santo António, 4099-001 Porto, Portugal; u14115@chporto.min-saude.pt; 11 Serviço de Doenças Infeciosas, Unidade Local de Saúde Gaia Espinho, 4434-501 Vila Nova de Gaia, Portugal; tiago.pinto.teixeira@ulsge.min-saude.pt; 12 Serviço de Doenças Infeciosas, Centro Hospitalar de Setúbal, EPE, 2910-446 Setúbal, Portugal; bica.casella@gmail.com; 13 Centro Hospitalar do Baixo Vouga, EPE, 3810-164 Aveiro, Portugal; caspsoeiro@gmail.com; 14 Serviço de Medicina, Centro Hospitalar Barreiro Montijo, EPE, 2830-003 Barreiro, Portugal; andreiameseiro@hotmail.com; 15 Serviço de Medicina, Centro Hospitalar Universitário do Algarve EPE—Unidade de Portimão, 8000-386 Portimão, Portugal; raquelbp5@hotmail.com; 16 Serviço de Medicina, Hospital Vila Franca de Xira, EPE, 2600-009 Vila Franca de Xira, Portugal; andreimmachadoribeiro@gmail.com

**Keywords:** people living with HIV, clinical care continuum, rapid ART initiation, retrospective analysis

## Abstract

**Background/Objectives**: The timely initiation of antiretroviral therapy (ART) in persons living with HIV (PLWH) can improve clinical outcomes. However, ART commencement is often delayed. Portugal, despite having one of the highest new HIV diagnosis rates within the European Union, has limited available national-level data. Prior evidence from 2017 to 2018 suggests that the average time to ART initiation exceeds the recommendations for optimal patient benefits. This study aimed to determine the number of days from the first hospital appointment to the commencement of ART among newly diagnosed PLWH in Portugal between 2017 and 2022 at the national level and across different hospitals. It was hypothesized that newly diagnosed PLWH in Portugal experience a delay in ART initiation beyond the recommended timeframe. **Methods**: A retrospective analysis of records from Portuguese public tertiary care hospitals, which manage most HIV patients, was conducted. Descriptive statistics (measures of central tendency, dispersion, and frequency) were applied, along with association tests and a binary logistic regression model to examine factors influencing the timing of ART initiation. **Results**: A total of 2229 cases (out of 3434 received) from 19 hospitals were considered eligible. The median time interval between the first hospital appointment and ART initiation was 29.00 days, with a decreasing tendency between 2017 and 2022. Patients initiating therapy after 14 days had higher CD4 levels and lower viral loads compared to those starting within 14 days, with statistical significance. **Conclusions**: Continuous and regular monitoring of key indicators, such as the time to ART initiation, is pivotal for assessing the effectiveness of HIV treatment programs and pinpointing areas in need of improvement.

## 1. Introduction

The benefits of early antiretroviral therapy (ART) for persons living with HIV (PLWH) at HIV diagnosis, regardless of their CD4 cell count or clinical stage, are well-documented in the literature [1,2,3,4,5,6,7,8,9,10,11]. These gains include improvement across a wide range of clinical outcomes, such as increased viral suppression and decreased onward HIV transmission, enhanced quality of life, and the prevention of AIDS- and non-AIDS-related morbidity and mortality. Early-start ART is especially valuable in settings where long delays are common due to extensive patient preparation before treatment initiation [2,8,12,13,14,15,16,17,18,19,20]. Same-day ART initiation is advocated by the World Health Organization (WHO) as a safe and effective strategy that can help to control the HIV epidemic [14,17,18,21,22]. Indeed, rapid ART initiation can play a crucial role in achieving the United Nations’ 95-95-95 targets for the HIV care cascade, which aim for 95% of all PLWH to be aware of their HIV status, 95% of diagnosed individuals to receive continued ART, and 95% of those on ART to achieve viral suppression [21,23].

Despite the benefits of early ART initiation, the HIV continuum of care encompasses several critical stages (i.e., awareness of symptoms, testing, diagnostic confirmation, linkage to care, clinical evaluation, and treatment initiation), each with barriers that may cumulatively delay ART initiation by weeks and significantly impact clinical outcomes [21,24]. These barriers can arise from various factors, including a limited knowledge and understanding of HIV/AIDS (which can lead to engagement in risky behaviors and unawareness of the onset of concerning symptoms), stigma and discrimination that can deter individuals from seeking testing and treatment, overburdened healthcare systems, lengthy waiting times, geographic barriers, sociodemographic and economic disparities, or management of co-infections or comorbidities [25,26,27,28].

Several studies have demonstrated the potential of immediate ART initiation to shorten the time to viral suppression and improve retention in care among patients with early clinical HIV diseases [13,14,15]. Moreover, some countries have successfully implemented strategies to reduce the time from diagnosis to ART initiation, leading to significant benefits in treatment and prevention outcomes at the community level [29,30,31].

Time to ART initiation is a pivotal operational measure that can be used to assess current healthcare performance and guide quality improvement efforts focused on streamlining the way services are organized and delivered at the health facility level and the system level [32,33,34,35]. Developing effective healthcare practice means more than reporting this measure yearly—it requires continuous monitoring at short time intervals to more accurately assess the impact of interventions and identify opportunities for improvement [35,36].

Portugal has one of the highest rates of new diagnoses of HIV infection and acquired immunodeficiency syndrome (AIDS) incidence in the European Union [37]. A national guideline issued in 2015 outlines the steps and the timeframe for the referral process after a reactive HIV test, although the maximum time between the first hospital appointment and ART initiation is not defined by the guideline [38]. A previous one-time evaluation of the time interval between HIV diagnosis and ART initiation in newly diagnosed patients, between 2015 and 2017, showed a mean treatment initiation delay of 76 days in 2015, decreasing to 69 and 68 days in 2016 and 2017, respectively [39].

Recognizing the critical importance of monitoring time to ART initiation, a pilot project was conducted in 2021 to set a national strategy for measuring time to ART initiation and demonstrate its relevance as a key performance indicator. The initiative covered the analysis of secondary data for the years 2017 and 2018 (the period shortly after the implementation of the national guideline of 2015), and encompassed 80% of newly diagnosed PLWH linked to care in Portuguese hospitals during that period. Findings indicated that ART commencement less than 14 days after the first hospital appointment only took place in 38.0% of the cases, while the median time until ART initiation was 21 days [40]. Although this represented the first systematic effort to start monitoring the referral process in Portugal, some reported data were incomplete or inaccurate.

Building on this foundation, a broader approach was implemented in 2022, covering a more extended timeframe of five years (2017–2022); a larger number of hospitals to include a wider national scenario of the entire HIV referral network for HIV infection in Portugal; and assessing time to ART initiation after the COVID-19 pandemic. The relevance of this new study lies in the novel data generated as there is a lack of regular monitoring of this type of information in the Portuguese context. Moreover, given that Portugal has a healthcare system that guarantees universal access to ART, assessing the impact of system-wide guidelines and policies on ART initiation timelines and associated delays throughout the process can provide valuable insights for settings with similar centralized healthcare infrastructures for HIV (and PLWH).

In this light, the results of the current study provide valuable lessons for healthcare policymakers and HIV care providers worldwide, especially those working in publicly funded healthcare settings aiming to improve time-sensitive interventions for HIV treatment.

Against this background, we aimed to (i) assess the time interval between the first hospital appointment and ART initiation among newly diagnosed PLWH in Portugal during the period spanning from 2017 to 2022 at the national level and across different hospitals within the country; (ii) identify potential variations in the time to ART initiation across different hospitals and patient characteristics; (iii) examine the trend in the time to ART initiation over time.

## 2. Materials and Methods

A retrospective analysis of records from Portuguese public tertiary care hospitals responsible for managing the care of most PLWH at Portugal’s mainland jurisdictional (country) level was conducted. Only cases of individuals aged 15 and older who were diagnosed with HIV—with available data regarding the first hospital appointment between January 2017 and March 2022 and ART initiation occurring within twelve months of the first appointment—were included. Patients for whom the date of HIV diagnosis or ART initiation could not be determined, patients who initiated treatment before the first appointment, or who had inconsistencies (which could not be resolved) in their reported data were excluded.

### 2.1. Data Collection

The 25 major public referral hospitals for HIV treatment in mainland Portugal were invited to participate in this study (census approach). In Portugal, HIV treatment is primarily offered within the public healthcare system, including specialist hospitals designed for HIV care, and is fully covered by the National Health Service (NHS). Private hospitals are not generally involved in the treatment of notifiable infectious diseases, including HIV. Individuals newly diagnosed with HIV are referred to public healthcare facilities within the NHS for treatment, ensuring a streamlined process for access to care. After contacting the HIV department directors of each hospital by phone or email, a face-to-face meeting was held to discuss the study details for those who agreed to participate.

After confirming willingness to participate, formal authorization was obtained from the ethics committee of each hospital before data collection began. A structured data collection form was distributed by email and sent to the department directors. Data were collected by one physician from each of the participating hospitals, manually checked for accuracy against the original clinical notes and records at the time of extraction (by the same physician), and then anonymized before being sent to one designated member of the research team by email. The anonymized data were referred to by the generic names “Center A”, “Center B”, etc., to avoid comparisons between centers with different structures.

### 2.2. Variables

The following data for each patient were manually extracted from hospital records: (i) date of HIV diagnosis; (ii) date of the first visit to a specialized HIV medical care center; (iii) date of ART initiation (i.e., date of ART prescription and/or collection); (iv) first TCD4+ count (initial evaluation within the first hospital visit); (v) viral load (initial evaluation within the first hospital visit); (vi) age at the time of commencement of ART; (vii) country of birth; (viii) sex; (ix) age; and (x) source of referral (inpatient or non-governmental organization [NGO] referral).

For the time-based indicators, the time from the first appointment to the commencement of ART was calculated as the number of days between the date of the appointment and the first day of ART initiation. A threshold of 14 days was established to distinguish between early vs. delayed ART initiation, following previous evidence [12,17,19,20,41].

The stage of HIV was categorized into three groups—A, asymptomatic; B, symptomatic; and C, late-stage HIV—adapting WHO’s clinical staging of HIV disease [27] and the classification adopted by the Directorate-General of Health [42], excluding the first stage of acute infection. The type of virus was classified into three groups: cases of HIV-1 infection, cases of HIV-2 infection, and cases of HIV-1 and HIV-2 co-infection [43,44]. HIV-2 tends to be more benign and with fewer pathogenic consequences compared to HIV-1; however, the first is also less common and naturally resistant to non-nucleoside reverse transcriptase inhibitors. For these reasons, a stratified analysis may provide relevant information regarding infected individuals [45,46].

### 2.3. Data Analysis

To ensure the accuracy of the analyses, data cleaning procedures were implemented to identify and eliminate inconsistencies or errors. These included removing the duplicate entries, records with missing or invalid values, and cases not meeting the study’s inclusion criteria. Specifically, entries were excluded if they had (i) a first hospital appointment date before 2017 or after 2022; (ii) an ART initiation date on the same day as or before the first hospital appointment, or more than six months after that; and (iii) evidence of a prior HIV diagnosis. Inconsistencies related to clinical data were also addressed. In addition, potentially identifiable variables were recoded using unique identification codes, and the same nomenclature was used for the same information to ensure consistency.

Data analysis was performed using SPSS Statistics for Windows, version 26.0. Descriptive statistics were used to summarize the data. For continuous variables, measures of central tendency (medians, and percentiles 25 and 75) and dispersion (standard deviation or interquartile range) were used. For categorical variables, the absolute number of individuals per category and respective percentages were determined. To assess associations between categorical variables, Pearson’s Chi-Square test was employed. When expected frequencies in contingency tables were below 5, the Fisher–Freeman–Halton exact test was applied. For comparisons of non-normally distributed continuous variables (viral load and CD4 count) across two groups (time to ART initiation: ≤14 days vs. >14 days [12]), the Mann–Whitney U test was used. Normality of the continuous variables was evaluated using the Kolmogorov–Smirnov and Shapiro–Wilk tests. A significance level of *p* < 0.05 was considered statistically significant.

### 2.4. Ethical Considerations

This study was approved by the Ethics Committees of all participating hospitals.

## 3. Results

**Figure 1:**
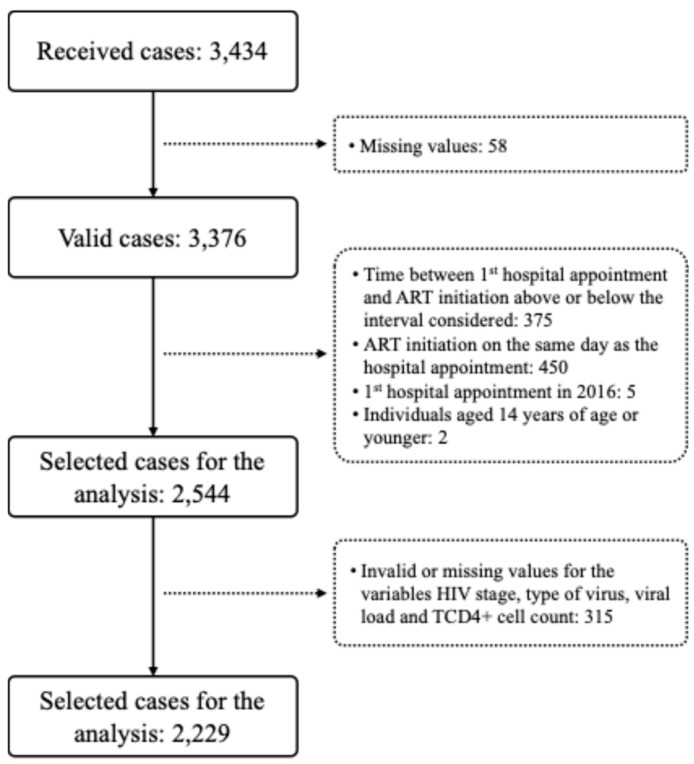
Study flowchart with the number of persons living with HIV whose cases were received, validated, and selected for analysis. Cases were excluded if they contained missing or invalid values or if they did not meet the inclusion criteria for this study.

From the 3434 cases received from the 19 participating hospitals, a total of 2229 (64.9%) were considered eligible. The total number of received and eliminated cases, by stage of data cleaning, is presented in Figure 1.

Overall, the median time interval between the first hospital appointment and ART initiation was 29.00 days (mean 39.00 days), with a range of 1 to 354 days (Table 1). A decreasing tendency is observed in the median time until ART initiation over the years, with the lowest median time in 2022 (18 days) and the highest in 2017 (35 days). More than two-thirds of the patients (77.0%) initiated ART more than 14 days after the first appointment.

A statistically significant association between HIV stage and the time interval between the first consultation and the initiation of therapy was found, showing that the distribution of time intervals varies according to disease stage. Patients who started therapy within 14 days of the first appointment tended to have lower TCD4 levels compared to those who started later. A statistically significant difference in viral load distributions between the two groups was also found as viral loads tend to be higher in the group that started therapy within 14 days compared to the group that started after 14 days (Table 2).

## 4. Discussion

The present study provides valuable insights into the time from first appointment to ART initiation among newly diagnosed PLWH in Portugal, spanning from 2017 to 2022. Overall, the median time to ART initiation was 29.00 days (almost four weeks), with a downward trend over the years, from 35 days in 2017 to 18 days at the beginning of 2022. This suggests an improvement in the timeliness of ART initiation over the past five–six years.

Moreover, in our study, patients with early ART initiation (14 days or less) after the first appointment had a higher viral load and a lower TCD4+ cell count, showing more advanced immunosuppression, which motivates earlier initiation of treatment to reduce viral load and the risk of transmission. This is particularly relevant as early start of ART for PLWH has already demonstrated well-established clinical benefits, including reduced risk of death, AIDS-related illnesses, serious non-AIDS illnesses, and sexual transmission of HIV due to behavioral modification, even though further research is necessary to determine the optimal timing of ART initiation [5,6,10,11,44,45]. While this study does not delve into the specific reasons, potential barriers to timely ART initiation may include inconsistencies in referral processes and protocols, unmet needs of specific populations, and psychosocial factors such as long wait times, inflexible appointment schedules, stigma, and an overburdened healthcare system facing high demand and limited response capacity due to healthcare worker shortages as doctors increasingly transition to private practice [25,26,27].

According to the European Centre for Disease Prevention and Control’s (ECDC) 2022 report, in most analyzed countries in the European Union/European Economic Area, 78% of the cases took only four days after HIV diagnosis until linkage to care, a number that increased to 98% for a linkage to care less than three months [47]. According to the ECDC’s report on HIV treatment and care, data from 2020 show that Portugal falls below (by less than 10%) the global 90-90-90 target in terms of the proportion of PLWH receiving ART [48]. Other studies have shown how different the realities in distinct countries are. For example, in Italy, a retrospective, observational study on newly HIV diagnosed patients from 2015 to 2019, the median time from the first care access to ART start was 24 days [49].

Our study included a sample of new HIV diagnosis that represents 35.5% of Portuguese cases between 2017 and 2022. However, national statistics entail all diagnosed individuals (either in Portugal or not) and not only those newly diagnosed, which makes us believe that our samples can be largely representative of the Portuguese scenario. This was possible due to the inclusion of a large number of hospitals responsible for managing the care of most PLWH.

The period under analysis in this study reveals a decreasing trend in the median time between initial hospital appointments and ART initiation, which was not altered after 2020 with the onset of the COVID-19 pandemic. Other authors have confirmed this finding, as the pandemic imposed several structural changes in healthcare services that prioritized acute clinical situations like newly diagnosed PLWH [23,50]. The COVID-19 pandemic was also accompanied by a concerning decline in the absolute number of newly diagnosed HIV cases in 2020 and 2021 (the year 2022 was not representative since this study only included the first three months). As reported by the ECDC, the pandemic’s disruption had a significant impact on HIV testing and reporting [47,51].

The observed heterogeneity in practices across the assessed centers underscores the need to examine and study existing practices to identify the causes of variability, particularly at a national level. While local sociological factors may contribute, it is essential to investigate the organizational factors at the institutional level that drive these disparities. The National Program for HIV/AIDS Infection could play a valuable role in leading efforts to understand these differences and implement targeted improvements.

A lower proportion of stage A and B cases in the ≤14 days group was also observed. This may reflect the clinical urgency associated with more advanced disease. Stage C cases were more frequent in this group (20% vs. 10% in the >14 days group), suggesting that patients with advanced HIV at presentation were prioritized for rapid ART initiation, as found in other studies [52].

Overall, the findings of this study highlight the progress that has been made in Portugal in enhancing the timeliness of ART initiation among newly diagnosed PLWH. These results have significant implications for the development of national and regional strategies aimed at improving the timeliness of ART initiation among newly diagnosed PLWH in Portugal. These strategies should focus on reducing variability in ART initiation timelines across different hospitals and enhancing access to ART for underserved populations, such as younger patients and those referred from NGOs. Additionally, the findings of this study suggest that the national guideline for the referral process for newly diagnosed HIV patients should be updated to include a clear definition of the maximum time between linkage to hospital care and ART initiation. This would aid in ensuring that all PLWH have access to ART as soon as possible following their diagnosis.

Although this is the first attempt to longitudinally monitor the time until ART start in Portugal, this study is limited by the retrospective nature of data collection. This means that some data may be missing or inaccurate. Moreover, data were gathered from hospital records—therefore, only covering the time after the first hospital appointment and until ART initiation—and excluded the following: data from the time of HIV diagnosis; cases where the diagnosis was established before the hospital appointment; cases of re-scheduled first appointments; and cases not assessing treatment adherence. Furthermore, around one-third of the cases were excluded after data cleaning, which may still introduce selection bias despite rigorous cleaning. Finally, out of the 25 hospitals invited to participate in this study, only 19 provided the necessary data. This limited participation may have hindered the ability to gather a more accurate picture of ART initiation for PLWH in Portugal. Even so, the authors are confident that the participating hospitals cover most new cases, which were included in this study [53]. Despite the efforts from the research team, several barriers have contributed to this, namely difficulties in obtaining responses from hospital management boards, a lack of cooperation from designated contact points for submitting this study to ethical committees, and instances where hospitals simply did not provide feedback after receiving the data request. Moreover, although all 19 hospitals provided the data on the core variables required for this study, differences in data completeness and reporting practices may have introduced some degree of information bias. Therefore, generalization of these findings to other populations should be approached with caution.

## 5. Conclusions

This study provides important information about the time from linkage to care to ART initiation in Portugal. The findings suggest that the time from hospital appointment to ART initiation is decreasing. However, there is still room for improvement, as advocated by the WHO. Continuous and regular monitoring and improvement of key indicators—such as the time to ART initiation—at short-time intervals is pivotal for assessing the effectiveness of HIV treatment programs and pinpointing areas in need of improvement. Future studies might explore patient-level drivers or the effect of simplified referral pathways.

An ongoing surveillance enables tracking progress, identifying potential bottlenecks in the care cascade, and implementing specific interventions to expedite ART commencement and optimize patient outcomes. Through consistent monitoring of these metrics, healthcare systems can ensure timely and effective treatment for PLWH, thereby reducing the risk of disease advancement and transmission.

## Figures and Tables

**Table 1:** Time (days) until ART initiation, over the years (n = 2229).

Year of the First Appointment	N	Median (IQR)	Mean (Standard Deviation)	Range (Minimum–Maximum)
2017	493	36.00 (33.00)	48.74 (48.15)	1–364
2018	497	30.00 (23.00)	39.54 (36.59)	1–309
2019	428	29.00 (30.50)	39.73 (42.07)	1–355
2020	348	21.00 (28.00)	36.62 (46.83)	1–351
2021	353	22.00 (23.00)	31.99 (39.08)	1–351
2022	110	21.00 (22.00)	26.15 (27.73)	1–222
Total	2229	29.00 (30.00)	39.30 (42.52)	1–354

Note: IQR, interquartile range.

**Table 2:** Clinical characteristics of patients on ART between 2017 and 2022 (n = 2229).

	Total N	≤14 Days n (%)	>14 Days n (%)	*p*-Value
HIV stage				
A	1512	316 (60.3%)	1196 (70.1%)	
B	458	104 (19.8%)	354 (20.8%)	**<0.001**
C	259	104 (19.8%)	155 (9.1%)	
Type of virus				
HIV1	2190	514 (98.1%)	1676 (98.3%)	
HIV2	3	1 (0.2%)	2 (0.1%)	0.902
HIV1 and 2	36	9 (1.7%)	27 (1.6%)	
Viral load				
Mean (SD)	756,175 (4,409,505)	1,1432,246 (4,927,177)	637,216 (4,232,190)	
Median (IQR)	83,100 (319,702)	138,000 (570,850)	70,718 (245,472)	**<0.001**
Minimum–maximum	0–101,000,000	37–100,000,000	0–101,000,000	
Initial TCD4+ cell count				
Mean (SD)	377.52 (331.06)	328.01 (257.29)	392.74 (349.28)	
Median (IQR)	338.00 (353)	292.50 (367)	353.23 (345)	<0.001
Minimum–maximum	1–9210	1–1362	2–9210	

Note: ER, emergency room; HIV, human immunodeficiency virus; IQR, interquartile range; NA, not available; NGO, non-governmental organization; SD, standard deviation. Bold font represents statistically significant values.

## Data Availability

The datasets analyzed during the current study are available from the corresponding author upon reasonable request.

## Appendix A

Portuguese HIV Study Group:
Pedro Gomes Santos: Serviço de Medicina, Centro Hospitalar Universitário do Algarve EPE—Unidade de Faro, Faro, Portugal.
Isabel Germano: Serviço de Medicina 1, Centro Hospitalar Universitário Lisboa Central (CHULC), EPE, Hospital de São José, Lisboa, Portugal.
Ricardo Correia Abreu: Serviço de Doenças Infeciosas, ULS Matosinhos, EPE, Hospital Pedro Hispano, Senhora da Hora, Portugal.
Isaac Pereira: Serviço de Medicina, ULS Baixo Alentejo, EPE—Hospital José Joaquim Fernandes, Beja, Portugal.
André Almeida: Serviço de Medicina, Centro Hospitalar Universitário Lisboa Central (CHULC), EPE, Hospital de Santo António dos Capuchos, EPE, Lisboa, Portugal.
Fausto Roxo: Unidade de Doenças Infeciosas, Hospital Distrital de Santarém, Santarém, Portugal.
Pedro Martins: Serviço de Doenças Infeciosas, Centro Hospitalar do Tâmega e Sousa, Penafiel, Portugal.
Raquel Diogo: Serviço de Doenças Infeciosas, Centro Hospitalar Universitário Lisboa Norte (CHULN), EPE, Hospital de Santa Maria, Lisboa, Portugal.
Gonçalo Cristóvão: Serviço de Doenças Infeciosas, Centro Hospitalar Universitário Lisboa Central (CHULC), EPE, Hospital de Curry Cabral, Lisboa, Portugal.
Carmela Piñeiro: Serviço de Doenças Infeciosas, Centro Hospitalar Universitário de São João (CHUSJ), EPE, Porto, Portugal.
Nuno Marques: Serviço de Infeciologia, Hospital Garcia de Orta, EPE, Almada, Portugal.
Ana Rodrigues: Serviço de Doenças Infeciosas, Hospital Professor Doutor Fernando da Fonseca, EPE, Amadora, Portugal.
João Rodrigues: Serviço de Doenças Infeciosas, Centro Hospitalar Universitário de Santo António, EPE, Porto, Portugal.
Joana Fragoso: Serviço de Doenças Infeciosas, Centro Hospitalar Vila Nova de Gaia/Espinho, Vila Nova de Gaia, Portugal.
Miguel Rodrigues: Serviço de Medicina, Centro Hospitalar de Setúbal, EPE, Setúbal, Portugal.
Filomena Freitas: Centro Hospitalar do Baixo Vouga, EPE, Aveiro, Portugal.
Daniela Rodrigues: Serviço de Medicina, Centro Hospitalar Barreiro Montijo, EPE, Barreiro, Portugal.
Liliana Pedro: Serviço de Medicina, Centro Hospitalar Universitário do Algarve EPE—Unidade de Portimão, Portimão, Portugal.
Carla Tonel: Serviço de Medicina, Hospital Vila Franca de Xira, EPE, Vila Franca de Xira, Portugal.
Álvaro Aires Pereira: Serviço de Doenças Infeciosas, Centro Hospitalar Universitário Lisboa Norte (CHULN), EPE, Hospital de Santa Maria, Lisboa, Portugal.
Vasco Almeida: Serviço de Doenças Infeciosas, Centro Hospitalar Universitário Lisboa Central (CHULC), EPE, Hospital de Curry Cabral, Lisboa, Portugal.
